# Obituary

**Published:** 1879-01

**Authors:** 


### OBITUARY.

Dr. Philip H. Austen.—Dr. Austen died in Baltimore, about three o'clock, October 28th, 1878. He was born in that City in 1822.

Gifted with a superior and enquiring mind, he made rapid progress in his studies and graduated from Yale College, in his nineteenth year. He next turned his attention to medicine and graduated with honors in his class. The practice of medicine was not congenial to his tastes and he soon abandoned it.

Making the acquaintance of Prof. C. A. Harris, he was induced to study dentistry, and at once entered the Baltimore Dental College. He stood at the head of his class. His deportment, artistic skill, superior mind and rare accomplishments at once attracted the attention of the Faculty. He graduated in the class of 1849. Close application to his studies so impaired his sight, that he soon gave up the operative branch of Dentistry. He was appointed to the chair of Dental Mechanism in the Baltimore Dental College in 1852. Naturally gifted, thoroughly trained to work, and splendidly cultivated as he was, (being master of seven languages), he believed himself unprepared, and at once determined to qualify himself for the untried duties. He gave up all other employment and toiled for three years before he was satisfied with his attainments as instructor. For the

College it was a most judicious and fortunate selection, as he was repeatedly called upon to teach anatomy, physiology and chemistry, and was never found wanting. For aptitude in imparting knowledge and understanding to his class he stood alone. No man could have excelled Dr. Austen as a teacher—he was simply perfection. When he retired from the College the loss was not to the College only, but an irreparable one to the Profession.

He made artificial Dentistry a specialty. His superior judgment, cultivated tastes, and great mechanical genius placed him in the foremost rank. His Christian character, integrity, and education refined and elevated this branch of Dentistry.

As Editor of Harris' Principles and Practice of Dentistry," (that department which received his special attention, was really original matter,) the numerous articles which he furnished for journals, and his translations of foreign works put him in the front rank as an author.

Dr. Austen's was one of the brightest and most thoroughly cultivated minds which have ever honored Dentistry.

He superintended and directed the working of the Austen Coal and Iron Mines, of Preston Co., W. Va., where he displayed great ability as an engineer and architect, and it has been said, had the company followed his practical advice and sound judgment, the mines would have proved one continued success.

We do not attempt a biography of the good and great man. This would require a volume. After this slight manifestation of high appreciation of the professional character, we must express our earnest and sincere esteem for the personal worth of the deceased. Friendship and the loving affection which has clustered around him for years justify it.

In our deep sorrow we are not inclined to murmur against the providence of God. We know the dispensations of Divine Wisdom are right. We submit, we retire into the sanctuary of our own heart and mourn, not too early for the fruition of his fame, not too early for an honored and beloved name.

Happy he, whose completed record shall bear as few defects, and as many virtues. He attained the highest earthly title, that of a Christian gentlemen; he held the mystery of the faith in a pure conscience.

In private life he was entertaining, pure in speech, fluent and earnest in conversation. He was pre-eminently free from guile and selfishness in both private and professional intercourse. No sharp practices, disingenuous advantages or mean hypocrisy disfigured or dishonored his private life or professional career. No impurity dimmed, no gall stained his soul. He cherished these virtues as an armor of refined gold, to be sacredly preserved through all the difficulties, trials and temptations of life.

He was all he seemed and professed to be—the natural man. He hated friendship not felt; he despised the pretender.

The sweetest charities and purest virtues were exemplified in his Christian life. Charity to his fellow man, justice to his neighbor, kindness to the poor, affection and honor in his home. In his family he was always cheerful and contented, and diffused the life-giving sunshine of his own sympathetic and unselfish nature. The life and character, the daily acts of such an one must always hang around the throbbing hearts of the bereaved and sorrowing.

Dr. Philip H. Austen is not dead—he still lives. C.—*Am. Jour. Dent. Science.*

